# The Generative Shortfall: a comparative analysis of autoregressive vs. extractive architectures in ABSA

**DOI:** 10.3389/frai.2026.1930455

**Published:** 2026-09-24

**Authors:** Chathurvedi V. Rama Chandra, T. Padmavathy

**Affiliations:** School of Computer Science and Engineering, Vellore Institute of Technology, Vellore, Tamil Nadu, India

**Keywords:** Aspect-Based Sentiment Analysis, class imbalance, edge computing, Generative Shortfall, QLoRA, Small Language Models, structural regularization

## Abstract

In natural language processing (NLP), Aspect-Based Sentiment Analysis (ABSA) remains a challenge, particularly when handling ambiguous, domain-specific “neutral” sentiment classifications in cases of severe class imbalance. While there has been a pivot toward deploying generative Small Language Models (SLMs) on resource-limited edge devices, their performance on unevenly distributed data remains under-explored. This paper presents a comprehensive, cross-paradigm evaluation of the following ABSA architectures: a sequence-based BiGRU, a state-of-the-art DeBERTa-v3 encoder, and Alibaba’s Qwen2.5-1.5B generative SLM. To evaluate the feasibility of deploying the SLM on edge devices, we modified the Qwen2.5 model to work on a 6GB VRAM device using BFloat16 quantized low-rank adaptation (QLoRA) and an abstract syntax tree (AST) parser. A closer look at the results shows a fundamental Generative Shortfall. Despite optimizing the SLM for edge devices, its performance was suboptimal (accuracy: 79.67%, macro-F1: 0.71), indicating that the model’s conservative token generation behavior limited its ability to identify sparse and subtle semantic targets within the text. While the DeBERTa-v3 encoder, when balanced with multi-sample dropout and layer-wise learning rate decay (LLRD), achieved a macro-F1 of 0.87, successfully identifying 81.95% of the minority neutral class. Supported by bootstrap resampling and McNemar’s statistical significance test ( p<0.001 ), the results show that although generative SLMs can be efficiently deployed on resource-constrained edge devices, structurally regularized extractive encoders provide significantly better performance for precise opinion mining on highly imbalanced data.

## 1 Introduction

As user-generated data increases, traditional methods for opinion mining are becoming less effective at capturing subtle meanings. In early sentiment analysis architectures, classification was mainly binary, assigning a positive or negative label to the data. This approach fails to capture the complex nature of real-world user feedback, where multiple and often completely opposing sentiments are expressed within the same text. Aspect-Based Sentiment Analysis (ABSA) emerged to address these limitations, by identifying different targets in a sentence and analyzing their sentiments independently.

Applying ABSA in the real world often comes with the issue of class imbalance. This is clearly shown in the multi-domain FABSA dataset, where “Positive” and “None” classes have more representation than the “Neutral” class (Kontonatsios et al., 2023). This imbalance makes it difficult for models to learn efficient decision boundaries for underrepresented classes. In these situations, traditional extractive models start showing weaknesses. Sequence models, such as Bidirectional Gated Recurrent Units (BiGRUs), are efficient in terms of computation, but highly prone to cross-polar leakage; they have difficulty identifying aspect-related cues within short sentences without being affected by unrelated sentiments from adjacent words. As described in (He et al., 2023), models like DeBERTa handle complex data more precisely by separating what is said from where it appears in a sentence using gradient-disentangled embeddings.

NLP has recently shifted toward generative Large and Small Language Models (LLMs/SLMs) that rely on autoregressive reasoning to overcome the limitations of extractive models. Full-parameter fine-tuning of these models incurs a huge amount of computational and storage costs, making them incompatible for resource-limited edge devices (Xu et al., 2024). To mitigate these constraints, there has been a shift toward Parameter-Efficient Fine-Tuning (PEFT) techniques, specifically Low-Rank Adaptation (LoRA). As proposed in (Hu et al., 2021), LoRA freezes the massive pre-trained weight matrices and injects trainable rank decomposition matrices into the transformer architecture. For edge deployment on 6GB VRAM consumer hardware, Quantized LoRA (QLoRA) further optimizes the efficiency and significantly reduces the memory footprint by backpropagating gradients through a 4-bit NormalFloat (NF4) quantized base model (Dettmers et al., 2023).

This study compares sequential, extractive, and autoregressive ABSA models to identify which architecture performs most reliably on highly imbalanced data. As the industry moves toward deploying quantized generative models to minimize compute costs, a critical issue remains unaddressed. It is currently not clear whether these generative models can resolve the strict minority class boundaries in real-world datasets, or if their step-by-step text generation process makes them less suitable for tasks that require identifying sparse and specific information.

Edge-optimized autoregressive SLMs struggle to handle complex, contextually imbalanced data unless task-specific adjustments are applied. The DeBERTa-v3 encoder, when balanced with Layer-Wise Learning Rate Decay (LLRD) and Multi-Sample Dropout, successfully prevents minority class collapse, achieving a Macro-F1 score of 0.87.

The three major contributions of this paper are:

1. Identifying the Generative Shortfall: Our results conclusively show that edge-optimized generative SLMs struggle with highly imbalanced ABSA tasks, often making overly conservative predictions and missing minority-class patterns.
2. Validating Generative Evaluation: Designed a bf16-quantized QLoRA framework combined with an Abstract Syntax Tree (AST) parser that suppresses hallucinations, enabling the execution and evaluation of Qwen2.5-1.5B on 6GB VRAM hardware.
3. Necessity for Architectural Regularization: When combined with regularization techniques such as Multi-Sample Dropout and LLRD, DeBERTa-v3 shows stable decision boundaries and better minority class sentiment detection.

## 2 Related work

### 2.1 Traditional deep learning approaches

Aspect-Based Sentiment Analysis (ABSA) has relied heavily on recurrent neural network architectures, such as Recurrent Neural Networks (RNNs), Long Short-Term Memory networks (LSTMs), and Bidirectional Gated Recurrent Units (BiGRUs), due to their ability to capture sequential dependencies and process temporal text data (Singh et al., 2025). To maximize these capabilities, Singh et al. (2025) proposed the OptiASAR model to improve the capture of long-term dependencies in healthcare reviews by integrating a hybrid BiLSTM-GRU architecture alongside NER-BERT embeddings. Jiang et al. (2024) proposed the Deep Context-Aware Sentiment Analysis Model (DCASAM) to enhance the ability to capture subtle contextual variations by combining a Deep BiLSTM with a Densely Connected Graph Convolutional Network (DGCN). Addressing the degradation of context across network layers, He et al. (2025) proposed the CABiLSTM-BERT architecture to completely preserve implicit feature representations from shallow and intermediate layers by routing pre-trained word vectors through a dual-layer BiLSTM. Ramasamy and Elangovan (2024) proposed the Optimized Cascaded Long Short-Term Memory (OCas-LSTM) framework to accurately predict sentiments from customer reviews by cascading LSTM networks and optimally tuning their parameters via a Modified Bird Swarm-Ladybug Beetle Optimization algorithm.

While these recurrent models improved sentiment classification, RNNs and LSTMs still have limitations, particularly in handling long-range dependencies and capturing aspect-related cues (Gracewell et al., 2025). Sequential models have difficulty finding sentiments that relate to particular elements in complicated sentences that have more than one opinion, and tend to pick up irrelevant context. To address these limitations, researchers have enhanced recurrent models with additional mechanisms to help them capture aspect-specific information. Gracewell et al. (2025) proposed the SCH-MGN model, which combines RNNs with semantic capsule networks to better capture complex sentiment cues. Nezhad et al. (2025) introduced the ElHBiAt model, which combines BiLSTM with cross-domain ELECTRA representations to avoid negative transfer. Wadawadagi et al. (2025) proposed the PDAN framework, which replaces aspect inputs with polarity embeddings before BiGRU processing to improve sentiment awareness. Despite these improvements, recurrent networks still have limitations due to their sequential nature. Their difficulty in capturing long-range dependencies emphasized the need for new approaches, leading to the rise of transformer-based encoders.

### 2.2 Evolution of transformer-based encoders

Recognizing the limitations of RNNs, research has shown that self-attention models are better at capturing context from both the directions without relying on sequential processing (Apostol et al., 2025; Devlin et al., 2019; Guo et al., 2025; Jia et al., 2025; Chauhan and Mohana, 2025). Unlike sequential models that struggle with long-range dependencies, pre-trained language models (PLMs) such as BERT, RoBERTa, and XLNet use self-attention to process the entire text at once, allowing them to capture context more effectively (Sun and Zhu, 2025; Verma et al., 2025; Wan et al., 2025; Yang et al., 2025; Zhu and Xu, 2025). By using contextual representations instead of simple word vectors, transformer encoders have addressed the limitations of earlier methods and become the foundation of modern ABSA tasks (Liu et al., 2025; Sun et al., 2024; Zhang and Xiang, 2025).

While PLMs are effective, self-attention treats all tokens similarly and can make it difficult to distinguish multiple aspects within a sentence, and may introduce irrelevant context. To effectively capture aspect-specific information, subsequent models added syntactic and semantic knowledge to the transformer (Jin et al., 2025; Li and Lei, 2025; Tian et al., 2025; Yang et al., 2025; Yu et al., 2025). These approaches combine Graph Convolutional Networks (GCNs) and Graph Attention Networks (GATs) with BERT to connect aspects with corresponding opinion words through dependency trees, semantic graphs and external knowledge (Guo et al., 2025; Jia et al., 2025; Liu et al., 2025; Wan et al., 2025; Zeng et al., 2025). Multimodal ABSA models also applied this technique by using cross-modal attention and visual filters to better link textual aspects with visual cues and filter out background noise (Zhu and Xu, 2025; Sun et al., 2024).

Although using BERT and graph neural networks introduces structural information, new models use DeBERTa to improve the handling of syntactic and positional relationships compared to previous encoders (Apostol et al., 2025). DeBERTa disentangles the content and position information in the attention scores, better capturing the syntax that was previously captured in GCNs. However, the computational requirements and model sizes of these encoders prompted the development of more efficient, smaller models, such as parameter-efficient generative approaches.

### 2.3 Parameter-efficient generative models and edge optimization

The growing trend in recent ABSA research has been to use generative Large Language Models and Small Language Models (LLMs/SLMs) to reduce the cost of large transformer models. Generative models (e.g., T5), and large LLMs (e.g., GPT-4), can process multiple sentiment elements by making sense of contradictory elements rather than enforcing classification borders (Aqeel and Setti, 2025; Cai et al., 2025; Dong and Wei, 2025). To fine-tune these models without the expense of full fine-tuning, Parameter-Efficient Fine-Tuning (PEFT) techniques like soft prompting and Low-Rank Adaptation (LoRA) are used (Zou and Wang, 2025).

With technological advancements in edge computing, efforts made in deploying generative models in edge-computing environments tends to be challenging because of their high memory requirements. Standard fine-tuning of large models often requires more than 24 GB of GPU memory, making it impractical on resource-constrained hardware with, for example, only 6 GB of VRAM. To address this limitation, Quantized Low-Rank Adaptation (QLoRA) reduces the memory requirements by storing the base model parameters in 4-bit NormalFloat (NF4) format while training low-rank adapter matrices to preserve the model’s ability to learn (Dettmers et al., 2023). This makes parameter-efficient fine-tuning possible for compact Small Language Models with around 1–2 billion parameters, such as Alibaba’s Qwen2.5-1.5B, even within strict edge hardware constraints.

Despite these compression techniques, recent studies on using LLMs and SLMs for ABSA tasks show an important difference between generative models and dedicated classification models (Esmaeel and Taqa, 2026). Large foundation models such as GPT-4 and GPT-3.5 can perform well in zero-shot settings, particularly when identifying general or implicit sentiment (Simmering and Huoviala, 2024). However, generative models can struggle to produce consistent predictions in specialized domains or highly imbalanced datasets when compared with supervised sequence encoders (Mughal et al., 2024).

Generative models may also produce inconsistent output formats or generate incorrect aspect–sentiment pairs, making their outputs more difficult to process reliably. Techniques such as domain knowledge decoupling and continual learning have therefore been explored to improve output consistency (Ding et al., 2024). Although unsupervised and prompt-based language models can capture broad semantic information (Eiras and Brito, 2026), they do not provide the same level of task-specific structure and regularization offered by dedicated extractive architectures.

Efficient generative models are particularly needed for processing diverse, real-world text. For this purpose, Setiabudi and Faizah (2025) introduced a BERT-based BiLSTM architecture for the FABSA dataset that achieved an accuracy of 93.34%. However, an analysis of this baseline shows that there is some room for improvement. Although the BERT model takes into account contextual information and the BiLSTM is good at capturing sequence-level information, the BERT-BiLSTM model is not good at ambiguous scenarios, such as the “Neutral” class in imbalanced data.

While prior work has explored Transformer Encoders and hybrid RNNs, little has been done to explore the effectiveness of edge-deployable SLMs on imbalanced data. In particular, there is no single framework to compare the performance of models such as Qwen2.5, fine-tuned using bf16 quantization and QLoRA to fit within VRAM constraints, with regularized extractive models in terms of the precision of the minority classes. This work addresses this gap by going beyond standard performance metrics and using statistical analysis to better understand whether generative models can be successfully applied to imbalanced opinion mining or if more sophisticated extractive models are still better suited for practical applications.

## 3 Materials and methods

### 3.1 Overview of the proposed framework

The aim of this research is to develop a cross-domain framework for Aspect-Based Sentiment Analysis (ABSA) and compare three distinct deep learning approaches. Traditional sentiment analysis methods are not well suited for complex user reviews that contain multiple, and often conflicting, opinions about multiple aspects. To address this, we propose an end-to-end pipeline consisting of (Figure 1):

1. Data Preparation: A domain-agnostic preprocessing step that converts complex, multi-aspect text data into structured mathematical targets.
2. Lightweight Extractive Baseline (BiGRU): A sequence model used as a baseline to evaluate efficiency and cross-polar leakage.
3. Transformer-Based Model (DeBERTa-v3): An extractive encoder that uses disentangled attention and regularization techniques to achieve strong “precision” performance especially on the minority class.
4. Edge Optimized Generative Model (Qwen2.5 via QLoRA): A generative approach to enable training and evaluation of a 1.5B parameter model on 6GB VRAM hardware using bf16 QLoRA.

**Figure 1:**
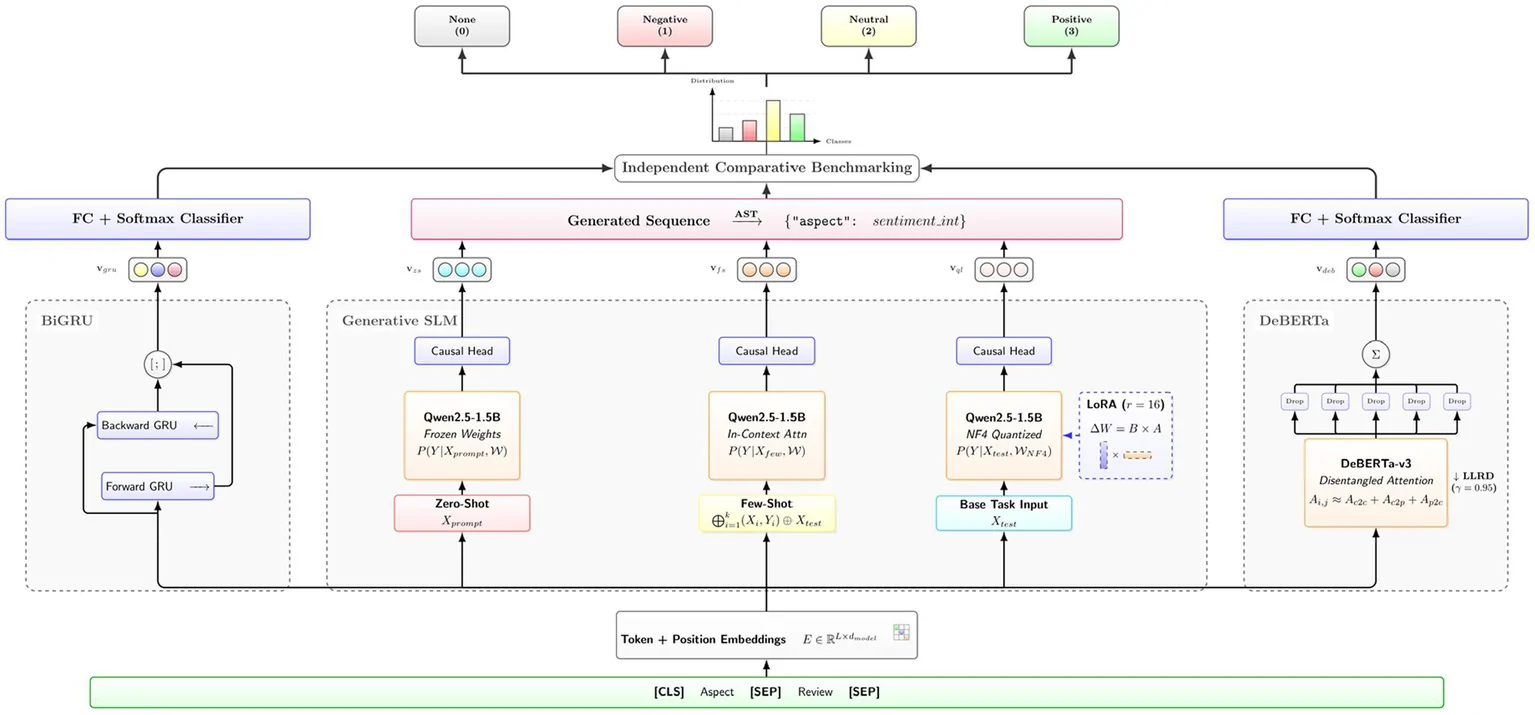
Proposed comparative architecture framework for Aspect-Based Sentiment Analysis.

### 3.2 Dataset description and corpus formulation

The evaluation was conducted using the FABSA dataset. Given the highly casual and noisy nature of the data, the data was pre-processed extensively before the training phase began. All text was normalized by converting it to lowercase and tokenizing it based on whitespace. For the extractive models, the text was mapped to a fixed vocabulary space, with a MAX_VOCAB_SIZE of 10,000 to remove less important tokens. Specific tensor indices for zero-padding (<PAD>) and unknown tokens (<UNK>) were also reserved. The generative SLM used its native Byte-Pair Encoding (BPE) tokenizer to preserve complex sub-word information, which is important for autoregressive generation.

#### 3.2.1 Domain diversity and data sources

The dataset contains user reviews from industries like fashion, travel booking, ride hailing, consulting, and groceries and were collected from mobile app platforms like Google Play Store and Apple App Store. This diversity in data helps prevent the models from overfitting to the language of a single industry and encourages them to learn more general aspect–sentiment patterns.

#### 3.2.2 Structural anatomy of the corpus

Each instance in the dataset is mathematically represented as a tuple $d_{i}=\langle {\mathrm{ID}}_{i},X_{i},\;Y_{i},\;C_{i},\;S_{i}\rangle \;$ , where:

1.
${\mathrm{ID}}_{i}$ : Unique numeric identifier.
2.
$X_{i}$ : Unstructured raw textual review (e.g., “*Easy to navigate. Crashes sometimes though*”).
3.
$Y_{i}$ : String-encoded list of aspect-polarity pairs (e.g., [‘online-experience.app-website.-1’, ‘purchase-booking-experience.ease-of-use.1’]).
4.
$C_{i}$ : Industry categorization.
5.
$S_{i}$ : Application source platform.

To translate unstructured text into a mathematical space, the FABSA dataset uses a hierarchical annotation scheme. Opinions are mapped using a Category. Attribute. Polarity framework.

Twelve unique aspect categories defined the evaluation space (e.g., company-brand. General-satisfaction, value. Discounts-promotions) and are mapped to a 4-dimensional classification target space: None (0), Negative (1), Neutral (2), and Positive (3).

#### 3.2.3 The class imbalance challenge

While the original FABSA dataset contains 12,696 unstructured user reviews, the raw text cannot be directly used for aspect-level classification. We used a hierarchical aspect-pairing approach by extracting the actual sentiments associated with the aspects mentioned in each review and applying negative sampling by adding up to three randomly selected aspects that were not mentioned in the text.

This process expanded the dataset to 50,285 aspect–text pairs and resulted in a highly imbalanced class distribution. The majority “None” class accounted for 31,725 instances (63.09%) and represented aspects that were not mentioned or were not relevant to a particular review. In comparison, the sentiment classes were less common: “Positive” contained 12,155 instances (24.17%), “Negative” contained 5,740 instances (11.41%), and “Neutral” was the smallest class, with only 665 instances (1.32%; Table 1).

**Table 1:** Class distribution and stratification of the FABSA dataset.

Sentiment class	Description	Total instances	Percentage of corpus	Imbalance severity
None (0)	No aspect mentioned/irrelevant	31,725	63.09%	Majority class
Positive (3)	Favourable opinion of aspect	12,155	24.17%	Minority
Negative (1)	Unfavourable opinion of aspect	5,740	11.41%	Minority
Neutral (2)	Ambiguous or mixed opinion	665	1.32%	Extreme minority

It is important to clearly distinguish between the two classes: “None” means that an aspect is not mentioned at all, whereas “Neutral” refers to an aspect that is explicitly mentioned but expresses a balanced or ambiguous opinion. Since sequential models process information step by step, they can struggle when strongly opposing sentiments appear in the same text. Information from one sentiment may influence the representation of another, making it harder for the model to preserve the correct local context.

#### 3.2.4 Dataset partitioning and stratification

To prevent data leakage between models and ensure robust evaluation, the 50,285 aspect-text pairs were divided using an 80:20 split. A single stratified test set containing exactly 10,057 samples was then used to evaluate all three models: BiGRU, DeBERTa-v3, and Qwen2.5. This ensured that each model was tested on the same data and that the distribution of all classes, including the minority “Neutral” class with 133 instances, was preserved. As a result, the models could be compared fairly using the same evaluation metrics and statistical tests.

### 3.3 Phase 1: The sequence modelling baseline (BiGRU)

#### 3.3.1 Contextual dense projection

BiGRU was used as a baseline to evaluate computational efficiency. Discrete tokens were embedded into a continuous space using frozen pre-trained weights. The resulting embeddings were then used as the input to the RNN.

To ensure reproducibility, we used a trainable embedding layer with 300 dimensions and two recurrent layers with a hidden size of 256 units. A spatial dropout rate of 0.3 was applied to reduce overfitting. The model was trained using the Adam optimizer with a learning rate of $1\times 10^{-3}$ for 10 epochs.

#### 3.3.2 Bidirectional gating formulation

To capture the interaction between an aspect term and its surrounding sentiment context, the sequence was processed bidirectionally. The model computed a forward sequence $\vec{h_{t}}$ and a backward sequence $\overset{\leftarrow }{h_{t}}$ . Both the hidden states were then concatenated as shown in Equation 1:

$$H_{t}=\vec{h_{t}}⊕\overset{\leftarrow }{h_{t}}$$ (1)

This concatenated vector was then passed through a fully connected layer to the ${\mathbb{R}}^{4}$ classification space.

### 3.4 Phase 2: Extractive state-of-the-art (DeBERTa-v3)

#### 3.4.1 Disentangled attention mechanism

DeBERTa-v3 (Decoding-enhanced BERT) was implemented to address the limitations of sequential information decay. Each token is represented by a content vector H and a relative position vector P . The attention score $A_{i,j}$ between token i and token j is the sum of three distinct projection matrices defined by Equation 2:

$$A_{i,j}\approx \underset{\text{content}-\text{to}-\text{content}}{\underset{︸}{H_{i}H_{j}^{⊤}}}+\underset{\text{content}-\text{to}-\text{position}}{\underset{︸}{H_{i}P_{ji^{⊤}}}}+\underset{\text{position}-\text{to}-\text{content}}{\underset{︸}{P_{\mathrm{ij}}H_{j}^{⊤}}}$$ (2)

This disentanglement is crucial for ABSA, allowing the model to distinguish between a sentiment modifier located two tokens away from an aspect versus ten tokens away.

#### 3.4.2 Structural regularization

To prevent overfitting of DeBERTa with more than 100 M parameters on a comparatively smaller FABSA dataset, we applied two regularization techniques:

1. Multi-Sample Dropout: The [CLS] token representation $h_{\mathrm{cls}}$ was passed through K=5 parallel dropout masks ( p=0.1 ). The final predicted probability $\hat{y}$ is the arithmetic mean of the outputs.
2. Layer-Wise Learning Rate Decay (LLRD): To preserve the lower-layer weights while fine-tuning the upper layers, learning rates were exponentially decayed from the top layer to the bottom. The learning rate for the k -th layer was defined as ${\mathrm{lr}}^{k}={\mathrm{lr}}^{\mathrm{top}}\cdot \xi^{L-k}$ , where ξ=0.95 .

### 3.5 Phase 3: Generative reasoning paradigm (SLM adaptation)

The final architecture shifts from extractive pattern matching to autoregressive text generation, using Alibaba’s Qwen2.5 (1.5B) (Qwen Team, 2025) to evaluate domain-specific sentiment reasoning.

#### 3.5.1 Zero-shot autoregressive formulation

Given the input text X and an engineered system prompt P , the SLM computes the probability of generating the exact sentiment token string S, as shown in Equation 3:

$$S:P(S\;|X,P)=\sum \prod_{i=1}^{|S|}P_{\theta }(s_{i}\;|\;s_{\left\{<i\right\}},X,P)$$ (3)

#### 3.5.2 Quantized low-rank adaptation (QLoRA)

Full-parameter fine-tuning of an SLM requires extensive memory, often exceeding the limits of edge devices. Keeping this in mind, the weights were compressed using 4-bit NormalFloat (NF4) quantization. Instead of updating the massive pre-trained self-attention matrices $W_{0}$ , LoRA injects low-rank decomposition matrices B and A, as shown in Equation 4:

ΔW=B×A (4)

where $B\in {\mathbb{R}}^{d\times r}$ , $A\in {\mathbb{R}}^{r\times k}$ , and rank r=16 . This reduces the number of trainable parameters by over 99%. The model also uses BFloat16 (bf16) precision. Unlike traditional PyTorch fp16 gradient scaling, bf16 preserves the range of weight updates while reducing memory, avoiding Out-Of-Memory (OOM) failures.

#### 3.5.3 Deterministic output mapping via abstract syntax trees (AST)

Generative models produce unrestricted text sequences, making them harder to evaluate classification tasks. We designed an extraction pipeline using Abstract Syntax Tree (AST) parsing. The model was prompted to format its output as a valid Python dictionary. The AST parser evaluated this generated string, filtered out hallucinated tokens and translated the output back into the ${\mathbb{R}}^{4}$ target space for metric comparison against the extractive models.

To reduce inconsistent or invalid outputs during autoregressive generation, we used a strict system prompt that required the SLM to produce its predictions in a predefined Python dictionary format. For the few-shot setting, three randomly selected examples were included directly in the prompt. During inference, a low temperature of 0.1 was used to reduce variation in the generated outputs and encourage consistent formatting.

The generated responses were evaluated using Python’s ast.literal_eval to safely parse the dictionary outputs produced by the model. To handle inconsistent or unexpected generations without disrupting the evaluation pipeline, we implemented a two-stage sanitization process. When a valid dictionary was generated, the parser retained the recognized aspect keys and converted the corresponding values to the required numeric format. If the model generated an alternative structure, such as a list or set, the parser converted the relevant items into the expected format. In instances such as incomplete brackets, invalid syntax, or unrecognized aspect keys, the fallback handler captured the error and assigned the prediction to the majority “None” class. Following QLoRA fine-tuning, syntax errors and invalid output structures were largely reduced. This indicates that the observed Generative Shortfall is primarily related to the model’s tendency to make overly conservative predictions rather than failures in parsing or output formatting.

### 3.6 Optimization objectives, hyperparameters, and evaluation metrics

To handle class imbalance when training the extractive models, we used weighted cross-entropy loss. By assigning a lower weight to the majority “None” class and higher weights to minority classes, the model focused more on differentiating sentiments rather than favoring the majority class. It is defined in Equation 5 as:

$$ℒ=-\frac{1}{N}\sum_{i=1}^{N}\sum_{c=1}^{C}w_{c}y_{i,c}\log(\hat{y_{i,c}})$$ (5)

where N denotes the batch size, C is the total number of target classes, $y_{i,c}$ represents the ground-truth binary indicator, and $\hat{y_{i,c}}$ is the predicted probability distribution.

To ensure proper training and comparison, hyperparameters were adjusted based on each model’s capacity. The DeBERTa-v3 model was trained for 8 epochs with a batch size of 32 and a base learning rate of 5e-5, using a cosine learning rate scheduler. The Qwen2.5 model was fine-tuned using QLoRA with a gradient accumulation step of 4 to simulate the effect of training with a larger batch size. To manage memory paging during weight updates, the optimization process used a learning rate of 2e-4 via the paged_adamw_8bit optimizer. The LoRA adapter was configured with an alpha (α) scaling factor of 32 and a dropout probability of 0.05.

Since highly imbalanced datasets can make a model appear accurate simply by predicting the majority class, overall accuracy alone is not sufficient for evaluating model performance. Macro-Averaged F1 (Macro-F1) was thus established as the primary evaluation metric. By calculating the unweighted mean of the F1-scores across all C classes, this metric ensures that minority classes equally impact the final evaluation, regardless of their size in the dataset. The metric is expressed in Equation 6 as:

$$F1_{\text{macro}}=\frac{1}{C}\sum_{c=1}^{C}2(\frac{\text{Precisio}n_{c}\cdot \text{Recal}l_{c}}{\text{Precisio}n_{c}+\text{Recal}l_{c}})$$ (6)

where ${\text{Precision}}_{c}$ and ${\text{Recall}}_{c}$ denote the precision and recall for class c , respectively.

### 3.7 Experimental setup and computational environment

To evaluate the resource efficiency of the generative model on the edge device, we used two different computational setups. The DeBERTa-v3 encoder, which is computationally intensive, was trained on a workstation equipped with an NVIDIA RTX A4000 GPU having 16GB of VRAM. Both the BiGRU and the quantized Qwen2.5 SLM were executed on a NVIDIA RTX 4050 GPU having 6GB of VRAM. All models used PyTorch, with the BitsAndBytes and PEFT libraries used for 4-bit quantization and low-rank adaptation.

### 3.8 Statistical variance and significance testing

Since performance metrics on highly imbalanced datasets can vary depending on the specific test samples, relying only on a single metric value may not provide a reliable comparison between models. To account for this variation without repeatedly retraining the models, we calculated 95% confidence intervals for Accuracy, Macro-F1, and Neutral F1 using non-parametric bootstrap resampling on the final test predictions. For each metric, n=1,000 bootstrap iterations were sampled with replacement from the 10,057 test set samples, utilizing a fixed random seed to ensure that the confidence intervals could be reproduced consistently.

To determine whether the performance differences between the extractive and generative models were statistically significant, we applied McNemar’s test. An independent *t*-test was not suitable because both models were evaluated on the same test dataset, meaning that their predictions were not independent. McNemar’s test is specifically designed to compare paired predictions from two models evaluated on the same set of samples.

To conduct the test, we constructed a 2×2 contingency table for the paired models, isolating the cases where they produced different results. The best-performing model DeBERTa-v3 was used as the control baseline and compared against BiGRU and the generative models. Let $n_{01}$ represent the number of test instances where the control model predicted correctly while the evaluated model failed, and let $n_{10}$ represent the instances where the evaluated model predicted correctly while the control failed. To avoid overestimation of statistical significance on our large evaluation set, we applied Edward’s continuity correction to the test statistic, defined in Equation 7 as:

$$\chi^{2}=\frac{{\left(\|n_{01}-n_{10}\|-1\right)}^{2}}{n_{01}+n_{10}}$$ (7)

The null hypothesis ( $H_{0}$ ) assumed that both models had approximately the same probability of making correct predictions, implying that $n_{01}\approx n_{10}$ . The resulting test statistic follows a Chi-squared distribution with one degree of freedom. A significance level of α=0.05 was used, and $H_{0}$ was rejected when the test indicated a statistically significant difference between the models.

## 4 Experimental results

The evaluation of the proposed models was performed on a stratified test set of 10,057 multi-aspect samples from the FABSA dataset. This dataset has a severe class imbalance: the “None” class makes up 63.09% of the samples, while the minority “Neutral” class constitutes a mere 1.32%. Completely depending on overall accuracy can be misleading as a model can achieve artificially high accuracy simply by predicting the majority class without capturing any semantic cues from the input data. Therefore, we focus on Macro-Averaged F1, class-wise Confusion Matrices, and Receiver Operating Characteristic (ROC) curves and 95% bootstrap Confidence Intervals (CI) to evaluate the performance on the minority class.

### 4.1 Extractive baselines: sequence leakage and contextual degradation

We begin by evaluating the Bidirectional Gated Recurrent Unit (BiGRU) as a baseline for pattern-based methods.

#### 4.1.1 Metrics and class-wise leakage

From Table 2 we can infer that, despite the low computation overhead, the BiGRU model achieved an overall accuracy of 81.34% and a Macro-F1 score of 0.72. However, observing various class predictions clearly reveals limitations of sequence models. As shown in Figure 2, the model suffers from severe cross-polar leakage, misclassifying a significant amount of Negative and Positive sentiments as “None.” Since BiGRU processes text sequentially, it struggles to isolate aspect-specific cues in dense reviews and is easily influenced by the adjacent irrelevant context.

**Table 2:** Class-wise performance metrics for the BiGRU model.

Sentiment class	Precision	Recall	F1-score	Support
None (0)	0.87	0.86	0.87	6,345
Negative (1)	0.55	0.63	0.58	1,148
Neutral (2)	0.68	0.62	0.65	133
Positive (3)	0.80	0.77	0.79	2,431
Accuracy			81.34%	10,057

**Figure 2:**
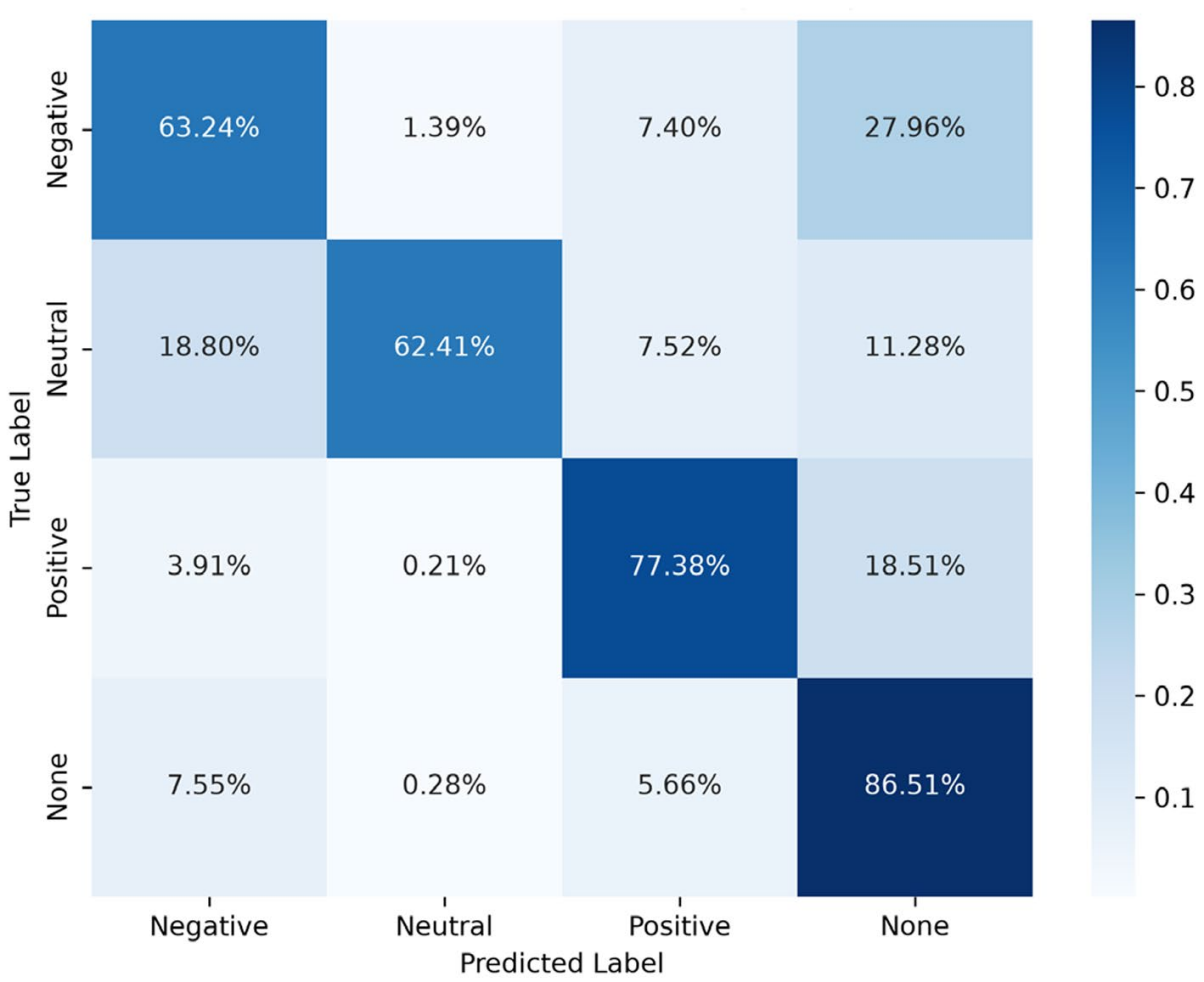
BiGRU normalized confusion matrix.

#### 4.1.2 Aspect-level precision and probabilistic thresholding

The model showed inconsistent performance when identifying ambiguous minority-class boundaries achieving a Neutral class recall of only 62.41% and a Neutral F1-score of 0.65 (95% CI: 0.58–0.72). The relatively wide confidence interval suggests that the model’s performance on the Neutral class can vary considerably depending on the test samples, making its predictions less reliable for applications where consistent performance is important.

Further analysis using the ROC curves (Figure 3) showed a similar pattern. While the model achieved strong AUC values for the majority “None” and “Positive” classes, its ability to distinguish minority-class semantic patterns was noticeably weaker.

**Figure 3:**
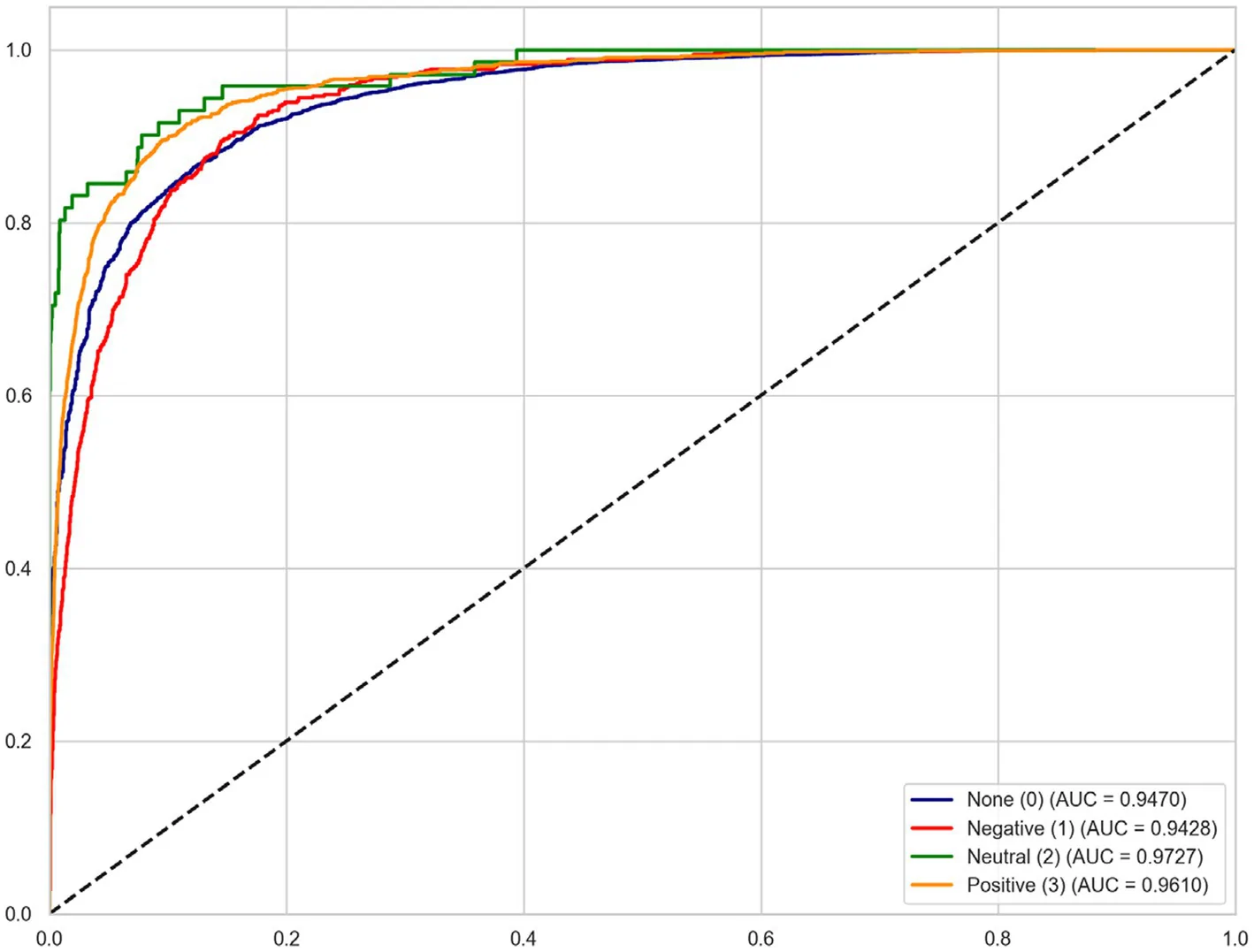
Multi-class ROC curves.

### 4.2 Deconstructing the “Generative Shortfall”

The quantized Qwen2.5-1.5B SLM was tested using a step-by-step setup: Zero-Shot, Few-Shot, and BFloat16 QLoRA fine-tuning.

#### 4.2.1 Zero-shot and few-shot failures

Under Zero-Shot prompting, the model achieved an accuracy of 64.21% (95% CI: 63.28, 65.14) and a Macro-F1 score of 0.35 (95% CI: 0.34, 0.36), failing to identify the minority Neutral class resulting in a neutral F1-score of 0.01 (Figure 4).

**Figure 4:**
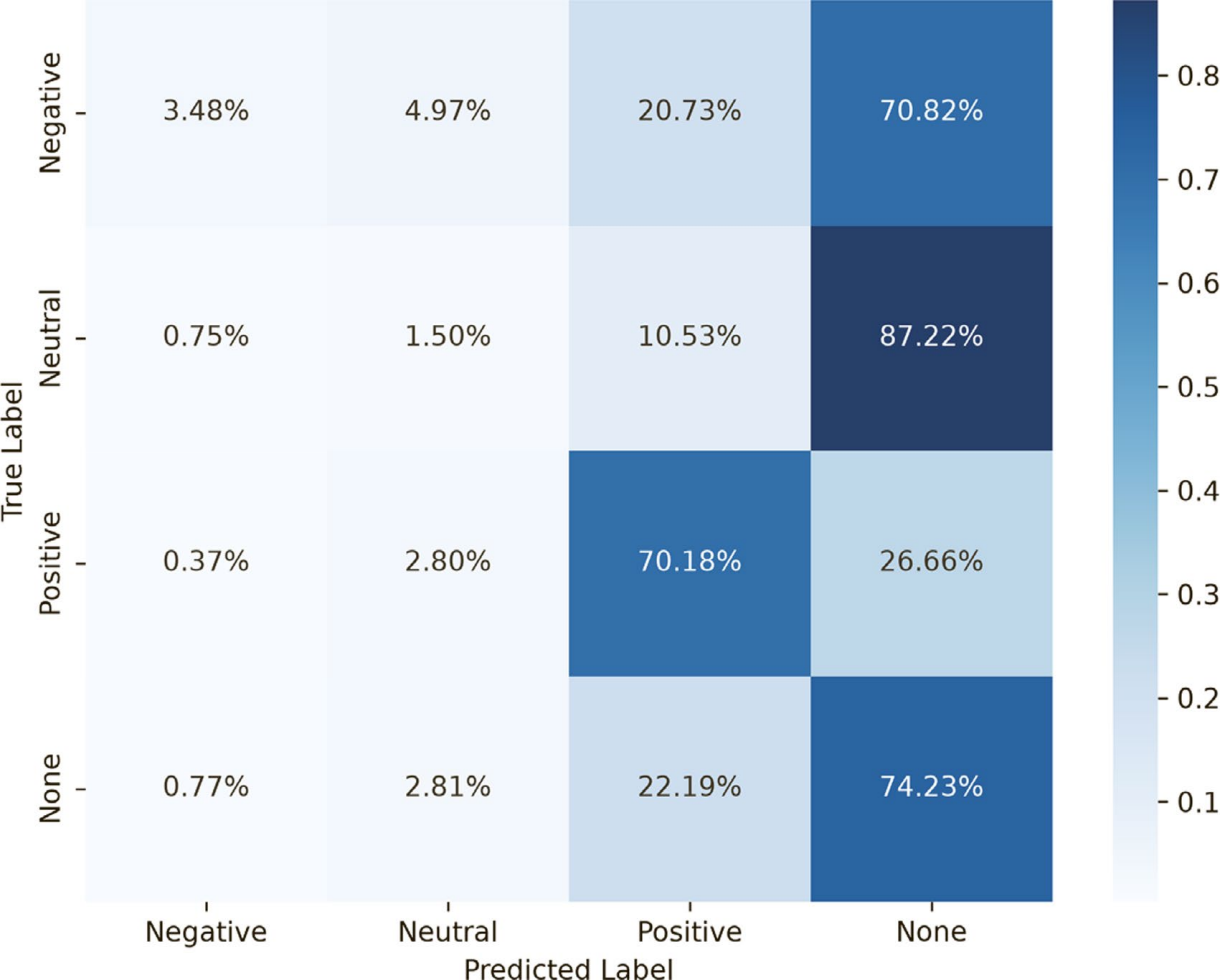
Qwen zero shot confusion matrix.

As shown in Table 3, the SLM performs very poorly when applied directly to the ABSA task fine-tuning or task-specific adaptation. Although the model achieves an global accuracy of 68.95%, this metric is influenced by the model’s ability to correctly predict the majority “None” class. The model completely failed to identify the minority “Neutral” class, resulting in an F1-score of 0.01 and Recall of 0.02. This shows that the model could not identify or separate aspect categories within the text. Thus, the results indicate that zero-shot generative models lack structural constraints and the necessary task adaptation for accurate and precise multi-aspect sentiment extraction.

**Table 3:** Class-wise performance metrics for Qwen2.5-1.5B under zero-shot prompting.

Sentiment class	Precision	Recall	F1-score	Support
None (0)	0.75	0.74	0.75	6,345
Negative (1)	0.40	0.03	0.06	1,148
Neutral (2)	0.01	0.02	0.01	133
Positive (3)	0.51	0.70	0.59	2,431
Accuracy			64.21%	10,057

Introducing Few-Shot prompting provided minimal additional context (Figure 5), marginally shifting the accuracy to 64.65% (95% CI: 63.70, 65.55). However, the Macro-F1 score dropped to 0.27. In both zero-shot and few-shot cases, the model failed to identify the minority “Neutral” class (Recall: 0.01; F1: 0.01), showing that the base models cannot capture domain-specific sentiment-cues, relying entirely on in-context learning.

**Figure 5:**
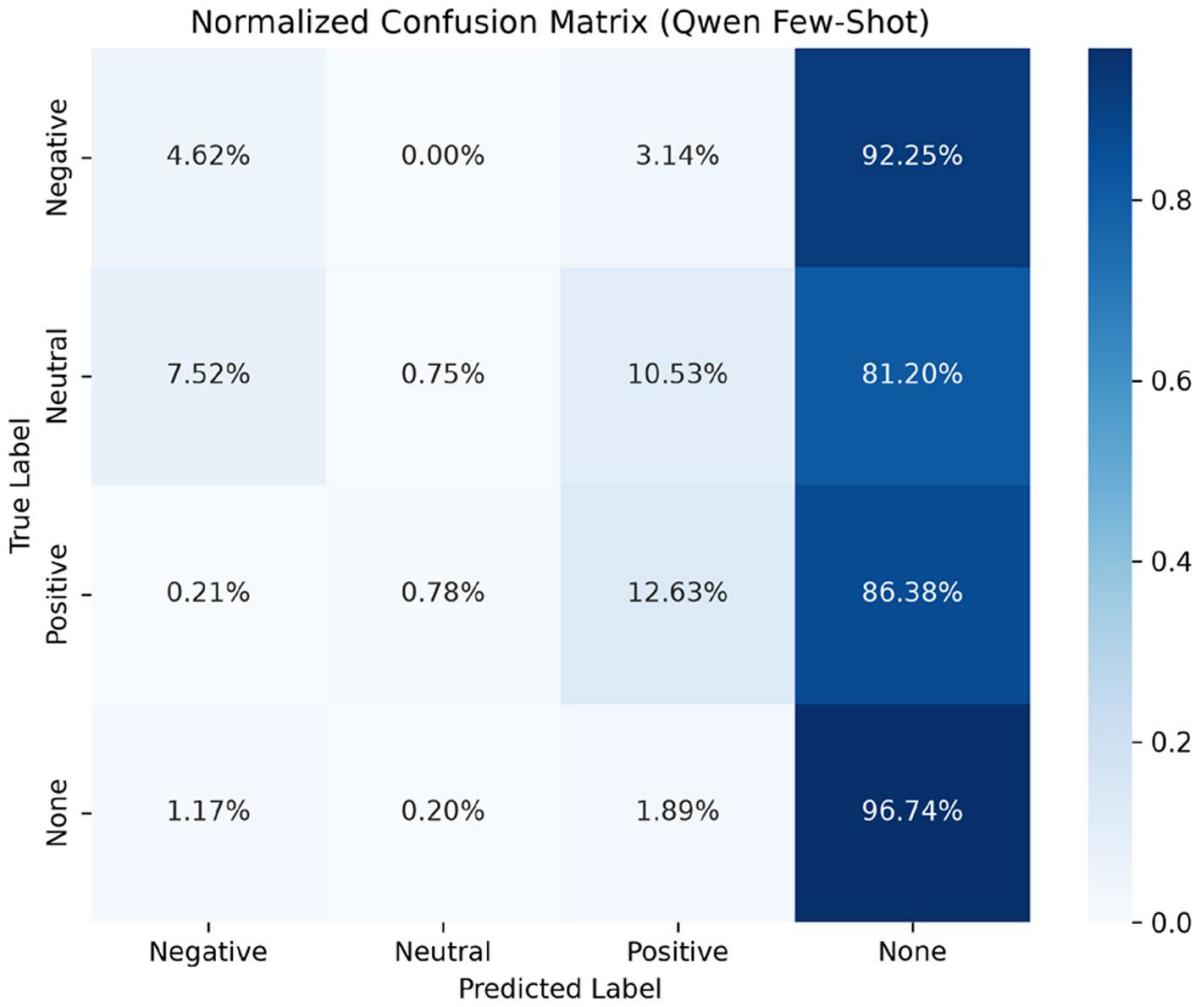
Qwen few shot confusion matrix.

Based on Table 4, while providing in-context examples slightly improved the consistency of aspect extraction, it also caused the model to rely more heavily on the majority class, leading to poorer performance on the active sentiment classes. The model achieved a high recall for the “None” class, due to it being the majority class. These results show that Few-Shot prompting does not provide enough task-specific learning capability to handle the imbalanced and complex ABSA semantics effectively.

**Table 4:** Class-wise performance metrics for Qwen2.5-1.5B under few-shot prompting.

Sentiment class	Precision	Recall	F1-score	Support
None (0)	0.65	0.97	0.78	6,345
Negative (1)	0.37	0.05	0.09	1,148
Neutral (2)	0.03	0.01	0.01	133
Positive (3)	0.64	0.13	0.21	2,431
Accuracy			64.62%	10,057

#### 4.2.2 QLoRA and generative conservatism

Applying QLoRA improved consistency of the model’s output. With AST-based structured parsing, the model achieved 79.67% accuracy (95% CI: 78.87, 80.45). Observing the results (Table 5) and normalized predictions (Figure 6) reveals the limitations of the autoregressive model when handling sparse data.

**Table 5:** Class-wise performance metrics for the fine-tuned Qwen2.5-1.5B (QLoRA) model.

Sentiment Class	Precision	Recall	F1-Score	Support
None (0)	0.82	0.99	0.89	6,345
Negative (1)	0.87	0.41	0.56	1,148
Neutral (2)	0.88	0.69	0.77	133
Positive (3)	0.94	0.48	0.64	2,431

**Figure 6:**
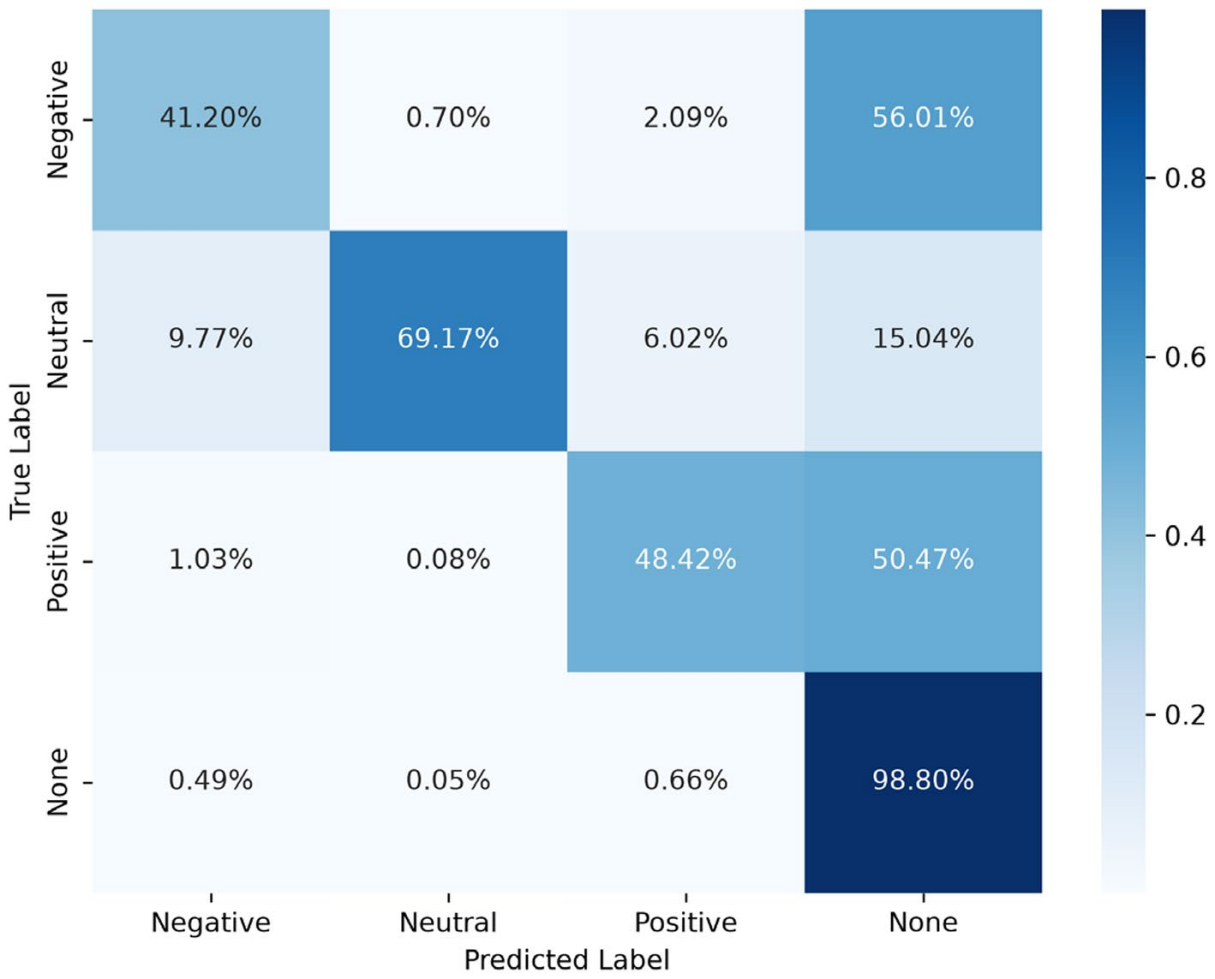
Qwen QLoRA confusion matrix.

Rather than accurately identifying active sentiment classes, the model became overly cautious and frequently defaulted to predicting the majority “None” class. As shown in the confusion matrix, this caused the model to miss 56.01% of true Negative aspects and 50.47% of true Positive aspects. In other words, the model incorrectly treated more than half of many valid sentiment targets as irrelevant or unmentioned.

We can infer from Table 5 that, applying BFloat16 QLoRA fine-tuning fixed many of the formatting and output consistency issues observed in the earlier approaches. However, the model achieved a Macro-F1 score of only 0.71 (95% CI: 0.69–0.73), which was still lower than the baseline BiGRU model (Macro-F1: 0.72). These results suggest that although generative SLMs can be efficiently compressed for edge devices, their step-by-step generation process makes them less suitable for accurately identifying aspects in highly imbalanced data.

Unlike extractive encoders, which produce continuous probability scores that can be used for variable-threshold analysis, the generative SLM produces discrete token outputs. Hence, traditional ROC analysis, which requires continuous prediction scores, cannot be directly applied to the generative model.

### 4.3 DeBERTa-v3 with structural regularization

To check whether a larger model could overcome the limitations of BiGRU and Qwen SLM, we evaluated the DeBERTa-v3 encoder.

#### 4.3.1 Convergence stability and regularization

Training large transformers on imbalanced data often leads to overfitting. However, as shown in the Loss Curves (Figure 7), using Layer-Wise Learning Rate Decay (LLRD) and Multi-Sample Dropout helped stabilize training. Both training and validation losses decreased smoothly without sudden spikes, indicating reduced overfitting to the majority class.

**Figure 7:**
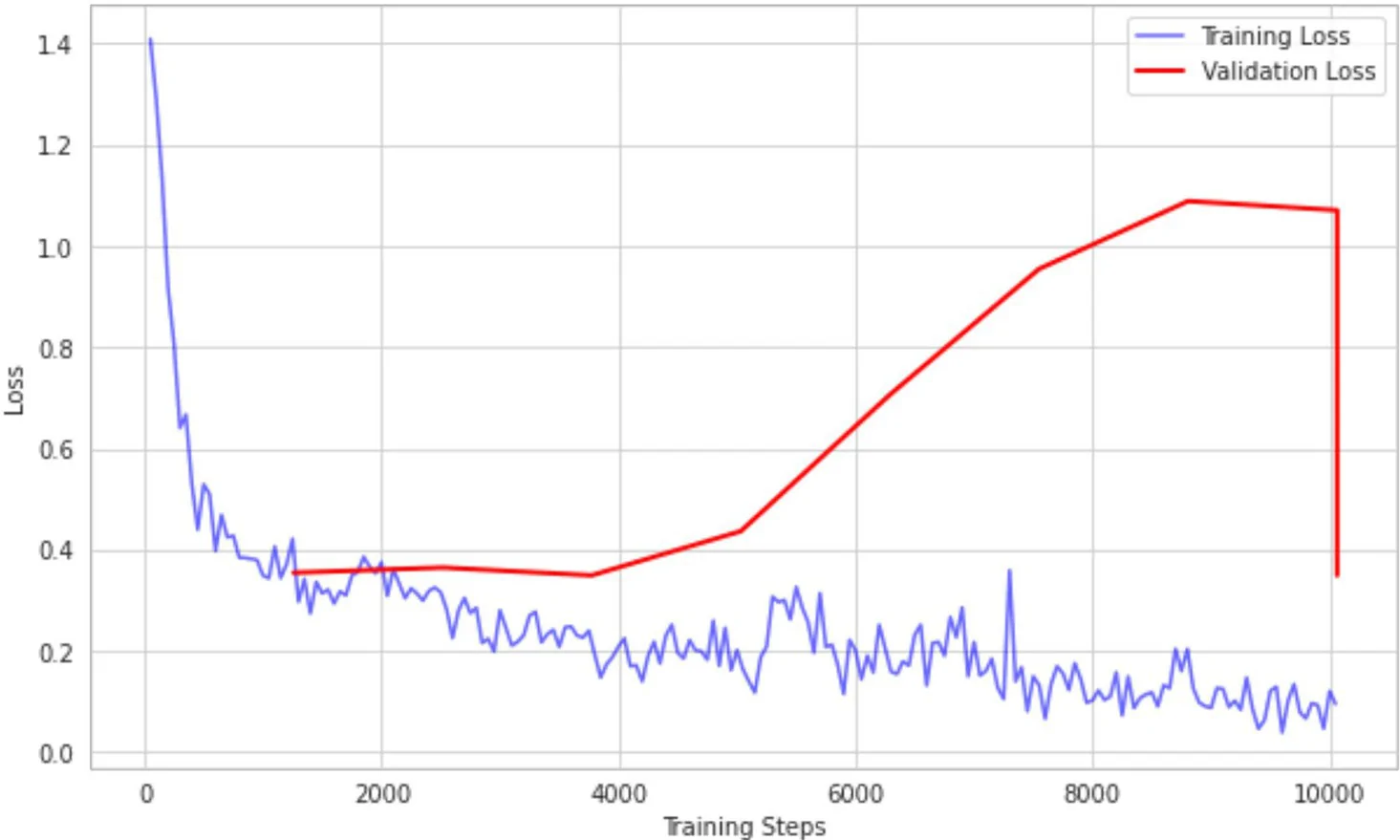
Training vs. validation loss DeBERTa.

#### 4.3.2 Improved performance on minority class

DeBERTa achieved Macro-F1 score of 0.87 and a Neutral F1-score of 0.81. As shown in Figure 8, it performed well on minority classes, correctly identifying 81.95% of Neutral and 84.84% of Negative instances.

**Figure 8:**
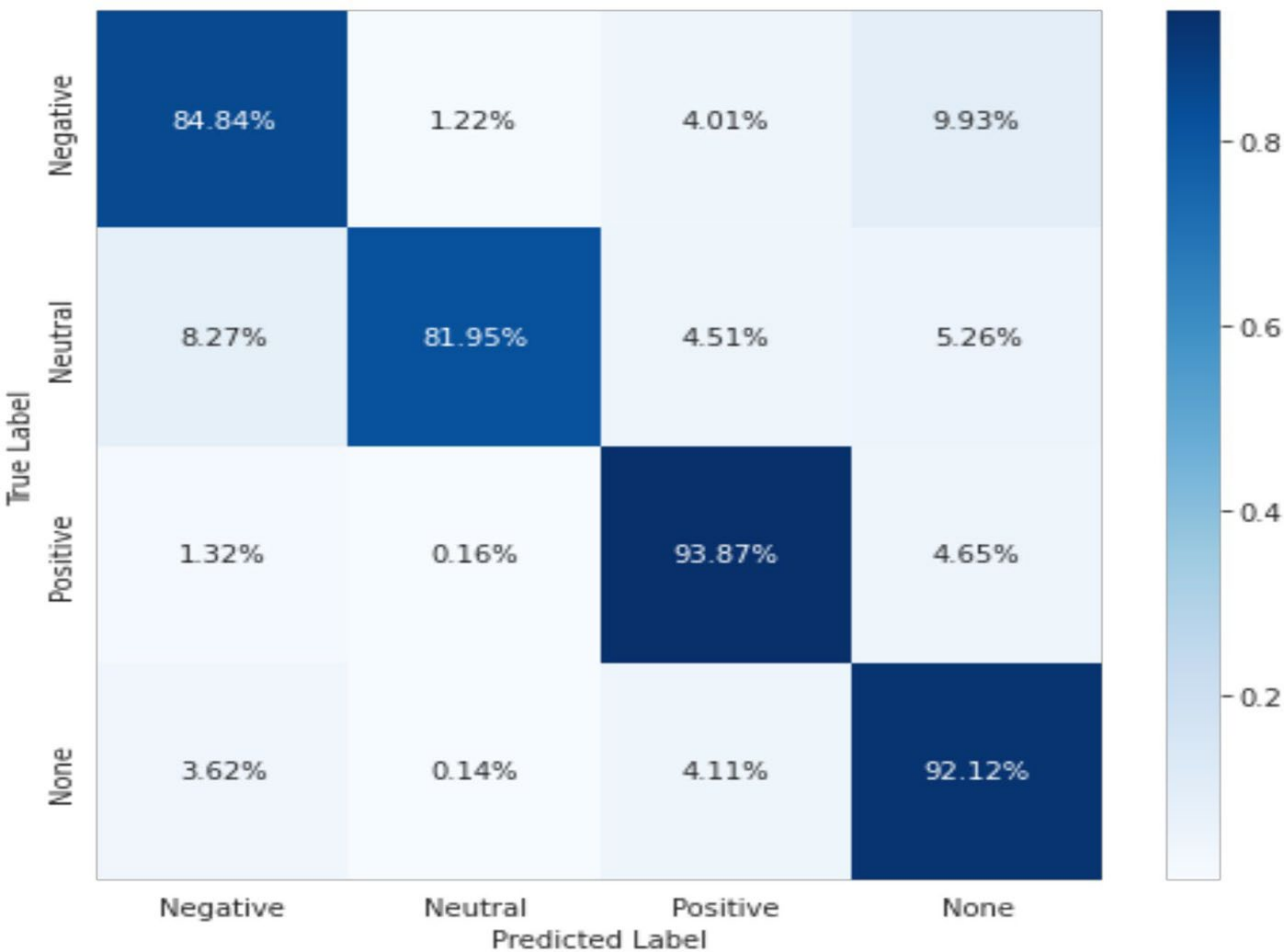
DeBERTa confusion matrix.

As shown in Table 6, the structurally regularized DeBERTa-v3 Base model, outperformed BiGRU and Qwen model in the comparative analysis with better performance metrics. The model demonstrated excellent aspect detection capability having a Neutral Recall of 0.82. It also was effective at classifying the minority class with the F1-score of the “Neutral” class at around 82%. Thus, for a highly imbalanced and multi-aspect sentiment analysis tasks, regularized extractive encoders are more reliable providing balanced performance.

**Table 6:** Class-wise performance metrics for the DeBERTa-v3 model.

Sentiment class	Precision	Recall	F1-score	Support
None (0)	0.96	0.92	0.94	6,345
Negative (1)	0.78	0.85	0.81	1,148
Neutral (2)	0.80	0.82	0.81	133
Positive (3)	0.88	0.94	0.91	2,431
Accuracy			91.58%	10,057

The ROC curves for DeBERTa (Figure 9) show close to perfect AUC scores across all classes, indicating consistent performance despite the class imbalance.

**Figure 9:**
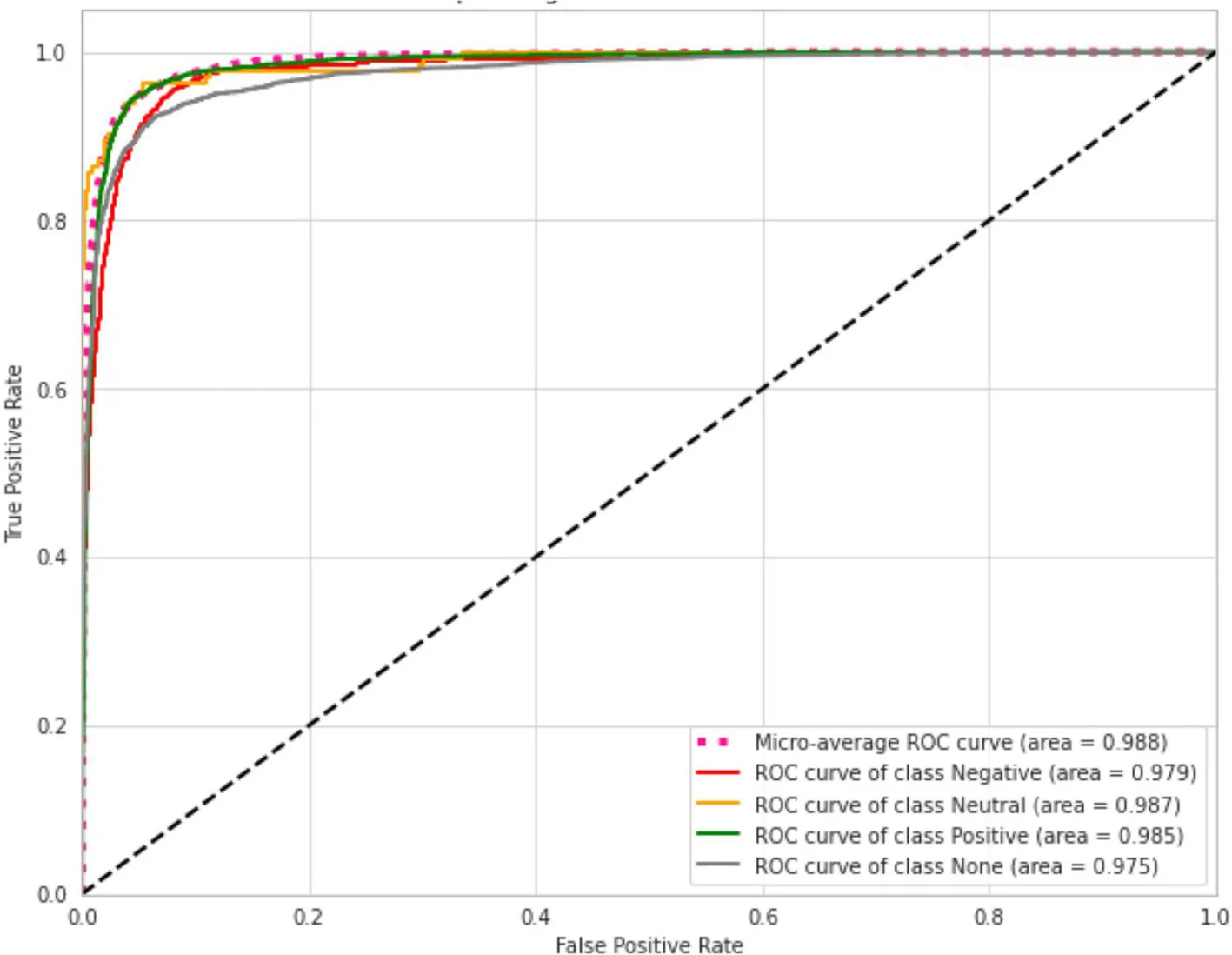
DeBERTa ROC curves.

### 4.4 Statistical comparison across models

To validate the performance differences between the sequential, generative, and extractive models, we applied statistical tests to their final predictions. Since the evaluation dataset contains a highly imbalanced class distribution, relying only on single performance scores may be misleading, as the results can be affected by variation in the test samples. Statistical testing was therefore used to determine whether the observed differences between the model architectures were reliable.

We used 95% Bootstrap Confidence Intervals ( n=1,000 resamples) to assess the stability of each model’s performance. To determine whether the performance differences between the models were statistically significant, McNemar’s test with Edwards’ continuity correction was also applied. The best-performing model, DeBERTa-v3, was used as the reference model and compared separately with the sequential and generative models. The null hypothesis ( $H_{0}$ ) assumed that the paired models had the same probability of making correct predictions.

The statistical analysis also highlights the limitations of the generative and sequential models when dealing with sparse sentiment targets. As per the results inferred from the Table 7, The Macro-F1 confidence intervals for DeBERTa-v3 ([0.85, 0.88]) and the QLoRA-adapted SLM ([0.69, 0.72]) do not overlap. This indicates the existence of a strong performance gap between the two models is consistent and unlikely to be caused by variation in the test samples. Also, it is inferred that the lower performance of the generative model reflects a more fundamental limitation of the architecture for this task.

**Table 7:** Statistical comparison and performance stability across models.

Architecture	Paradigm	Macro-F1 (95% CI)	Neutral F1 (95% CI)	McNemar’s χ2	Significance
DeBERTa-v3 (control)	Extractive	0.87 [0.85, 0.88]	0.81 [0.75, 0.86]	–	–
BiGRU	Sequence	0.72 [0.70, 0.74]	0.65 [0.58, 0.72]	1,029.00	p<0.001
Qwen2.5-1.5B (QLoRA)	Generative	0.71 [0.69, 0.73]	0.77 [0.71, 0.83]	695.01	p<0.001
Qwen2.5-1.5B (few-shot)	Generative	0.27 [0.26, 0.28]	0.01 [0.00, 0.04]	2,210.08	p<0.001
Qwen2.5-1.5B (zero-shot)	Generative	0.35 [0.34, 0.36]	0.01 [0.00, 0.02]	2,746.01	p<0.001

The McNemar tests with very large $\chi^{2}$ statistics across all comparisons, results in extremely small p -values (for example, $3.63\times 10^{-153}$ when compared with QLoRA). With a significance level of α=0.05 , the null hypothesis ( $H_{0}$ ) was rejected in all cross-model comparisons. These results confirm that the structurally regularized DeBERTa-v3 model performs significantly better than both the sequential and generative models when identifying and classifying complex aspects in highly imbalanced data and also does not imply a simple-small or random improvement.

### 4.5 Final verdict

The comparison of all three models is shown in Figure 10 and summarized in Table 8.

**Figure 10:**
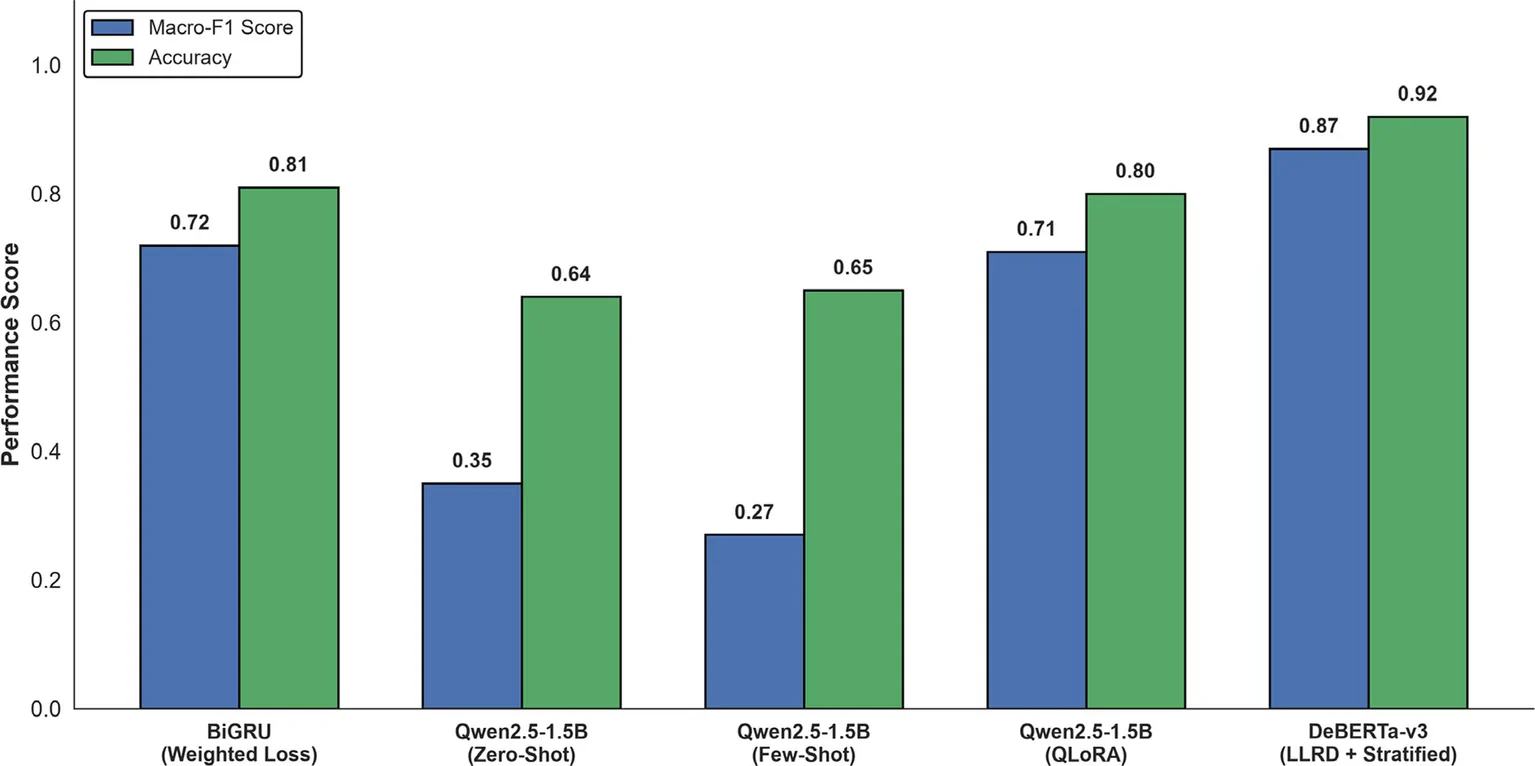
Performance comparison graph of BiGRU, DeBERTa and Qwen.

**Table 8:** Cross-paradigm performance comparison on the FABSA dataset.

Architecture	Paradigm	Accuracy	Macro-F1 score	Minority class (neutral) F1
BiGRU	Extractive (sequence)	81.34%	0.72	0.65
Qwen2.5-1.5B (Zero-Shot)	Generative (base)	64.21%	0.35	0.01
Qwen2.5-1.5B (few-shot)	Generative (in-context)	64.62%	0.27	0.01
Qwen2.5-1.5B (QLoRA)	Generative (PEFT)	79.67%	0.71	0.77
DeBERTa-v3 (LLRD)	Extractive (transformer)	91.58%	0.87	0.81

The comparison as shown in (Figure 10) supports the central hypothesis of this paper. While the Qwen2.5 (QLoRA) model achieves accuracy of 79.67%, its Macro-F1 score of 0.71 shows its limitations in handling nuanced sentiments. The DeBERTa-v3 encoder achieves similar accuracy (91.58%) but a much higher Macro-F1 (0.87). These results show that extractive encoders remain more reliable for opinion mining on imbalanced data.

Hence from Table 8 and Figure 10, we prove that the “Generative Shortfall” is a challenge that needs to be addressed while deploying autoregressive models on applications that deal with imbalanced data. Although they are memory efficient and are capable of executing on restricted hardware, they collapse while extracting the sentiment cues in the data. Even though extractive encoders take up compute and resources, they are a better alternative as they are efficient in capturing rare semantic cues with great precision and accuracy.

## 5 Discussion

A comparative analysis of ABSA models, evaluating BiGRU, DeBERTa, and Qwen2.5-1.5B on the FABSA dataset is presented, focusing on their ability to handle complex and conflicting opinions under class imbalance.

The evaluation reveals a problem in current generative models, which we formally define as the “Generative Shortfall.” We benchmarked an edge-deployable Small Language Model (Qwen2.5-1.5B) fine-tuned via BFloat16 QLoRA using an evaluation pipeline built on an Abstract Syntax Tree (AST) parser on restricted 6GB VRAM hardware. While this adaptation resolved formatting inconsistencies, it struggled with subtle semantic differences. The model defaulted to majority class biases, ignoring the active sentiment classes and yielding a Macro-F1 score of 0.71. Strikingly, despite the advanced parameter-efficient fine-tuning, the generative model failed to surpass the baseline sequence architecture (BiGRU Macro-F1: 0.72). This shows that while quantized parameter-efficient fine-tuning is effective on resource-limited edge devices, autoregressive SLMs remain inadequate for isolating complex, underrepresented sentiments.

### 5.1 The mechanics of Generative Shortfall

The difference in how extractive encoders and generative SLMs handle minority classes can be explained by their different optimization objectives. Extractive classifiers learn to predict the probability of a class based on the input, P(Y∣X) , from a fixed set of labels, C={0,1,2,3} . This makes it possible to use class-weighted cross-entropy to give greater importance to minority classes and reduce bias toward the majority class during training.

In contrast, autoregressive SLMs are trained to predict the next token in a sequence, optimizing the likelihood of each token based on the tokens that came before it, as shown in Equation 8:

$$P(S|X)=\prod_{i=1}^{|S|}P_{\theta }(s_{i}|s_{<i},X)$$ (8)

In highly imbalanced datasets where the unmentioned “None” class accounts for more than 63% of the samples, the model is exposed far more frequently to this majority class during training. As a result, during generation, predicting “None” often becomes the safest and most likely choice. The model may therefore favor the majority class instead of taking the risk of predicting less frequent classes, such as “Neutral,” which represents only 1.32% of the data. This creates a tendency toward overly conservative predictions and makes it more difficult for the model to identify minority sentiment classes.

### 5.2 Hardware constraints and real-world implications

The difference in hardware requirements highlights an important trade-off for real-world deployment. DeBERTa was evaluated on a workstation with 16 GB of VRAM, while Qwen was designed to run on a more resource-constrained 6 GB edge-grade GPU. Although QLoRA makes it possible to deploy Small Language Models on limited hardware, the resulting Generative Shortfall can have serious practical consequences. In high-stakes applications, such as financial forecasting or healthcare sentiment monitoring, repeatedly predicting the majority class may cause the model to miss important but less common signals. Furthermore, in social computing and customer feedback applications, this tendency toward conservative predictions can create a risk of overlooking underrepresented voices. Feedback expressed through less common dialects, language patterns, or specific concerns may appear less frequently in the data and therefore be more likely to be missed by the model. As a result, a system may achieve high overall accuracy while still failing to capture the opinions and concerns of smaller or less represented groups. Larger generative models, such as those with 7B or 13B parameters, may improve performance, but their increased memory requirements make them difficult to deploy on resource-constrained edge devices. These results highlight the trade-off between computational efficiency and reliable performance. For applications that require accurate identification of rare or subtle opinions, extractive encoders currently provide a more reliable option for localized opinion mining.

### 5.3 Future work

Despite structurally balanced extractive models currently performing better on imbalanced data, NLP will likely continue moving toward generative reasoning. Future research should therefore focus on improving how generative models handle minority classes in imbalanced data. One possible direction is data augmentation: oversampling the minority class data during the training phase to better balance the model. Other approaches, such as exploring contrastive alignment algorithms or hybrid neuro-symbolic prompting techniques may help generative models better distinguish between aspects and improve their precision, potentially closing the precision gap between generative and extractive encoders.

## Data Availability

The datasets presented in this study can be found in online repositories. The FABSA dataset is available at: https://github.com/kontonag86/fabsa-dataset. The code and training scripts are available at: https://github.com/chathurvedivanga/Generative-Shortfall-ABSA.
